# Glances at the Hospitals

**Published:** 1903-12-19

**Authors:** 


### GLANCES AT THE HOSPITALS.

### BEDFORD COUNTY HOSPITAL.

The receipts for the year amounted to £6,540, and the expenditure to £6,441, but this included the repayment of a sum of £1,307 due to the treasurer in the previous year, so that we may infer that the finances of the institution are in a satisfactory state. The total number of in-patients was 1,010, and of these 287 were medical, 614 surgical, and 109 ophthalmic. Of the total number 602 were discharged cured, 243 relieved, 35 not relieved, and 51 died. When the year closed 79 were in residence, and the average daily number resident was 73. The average duration of treatment was 26 days, the average cost was £4 12s. 2d., the average cost per bed was £63 15s. 3d., and the cost of provisions was 5s. 6d. a week. The number of out-patients was large, being 2,928, irrespective of 1,961 casualties. It is not without reason, therefore, that the board draw special attention of the Governors to this number, and they emphasise their request that the circumstances of all applicants should be carefully inquired into. As a matter of fact the out-patients' department of any hospital is very likely to be abused, and some hospitals have a wage limit above which gratuitous medical aid is refused. We notice that the work-people's own subscriptions amounted only to

£98, a sum which we believe ought to be considerably increased. The tables are carefully compiled, and the operation table shows the anæsthetic used as well as the results of the operation. Of 14 cases of hernia operated on, recovery followed in 13 and relief in one. There were 4 cases of ovariotomy and all recovered, and the same good result was obtained in 3 cases of abdominal hysterectomy. Altogether 636 operations were performed during the year. Of these cases 490 recovered, 112 were relieved, 3 were not relieved, and 10 died. Chloroform was given in 588 of the operations, ether in 7, A.C.E. mixture in 3, anestile in 1, cocaine in 33 and nitrous oxide gas in 2.

